# Immobilization in Ionogel: A New Way to Improve the Activity and Stability of *Candida antarctica* Lipase B

**DOI:** 10.3390/molecules25143233

**Published:** 2020-07-15

**Authors:** Alfonso Escudero, Antonia Pérez de los Ríos, Carlos Godínez, Francisca Tomás, Francisco José Hernández-Fernández

**Affiliations:** 1 Department of Chemical and Environmental Engineering, Technical University of Cartagena (UPCT), Campus La Muralla, C/Doctor Fleming S/N, E-30202 Cartagena, Murcia, Spain; alfonso.escudero@upct.es (A.E.); carlos.godinez@upct.es (C.G.); 2 Department of Chemical Engineering, Faculty of Chemistry, University of Murcia (UMU), P.O. Box 4021, Campus de Espinardo, E-30100 Murcia, Spain; aprios@um.es (A.P.d.l.R.); ptomas@um.es (F.T.)

**Keywords:** enzymatic immobilization, organogels, ionogels, ionic liquid, ester synthesis, enzyme, green chemistry

## Abstract

New *Candida antarctica* lipase B derivatives with higher activity than the free enzyme were obtained by occlusion in an organogel of an ionic liquid (ionogel) based on the ionic liquid [Omim][PF_6_] and polyvinyl chloride. The inclusion of glutaraldehyde as a crosslinker improved the properties of the ionogel, allowing the enzymatic derivative to reach 5-fold higher activity than the free enzyme and also allowing it to be reused at 70 °C. The new methodology allows enzymatic derivatives to be designed by changing the ionic liquid, thus providing a suitable microenvironment for the enzyme. The ionic liquid may act on substrates to increase their local concentration, while reducing water activity in the enzyme’s microenvironment. All this allows the activity and selectivity of the enzyme to be improved and greener processes to be developed. The chemical composition and morphology of the ionogel were also studied by scanning electron microscopy–energy dispersive X-ray spectroscopy, finding that porosity, which was related with the chemical composition, was a key factor for the enzyme activity.

## 1. Introduction

In recent decades, ionic liquids have demonstrated their potential for use as reaction and separation media. For example, in separation applications, they have been used as liquid–liquid biphasic systems [1,2] and as liquid phase in supported liquid membranes and polymer inclusion membranes [3,4,5]. In the field of biocatalysis, ionic liquids have been used as free solvent [6,7,8], adsorbed [9,10,11,12], covalently linked to particle enzymes [13,14,15], or as polymeric ionic liquids [16] in enzyme particles, in all cases to create an adequate microenvironment for the enzyme or to improve catalytic efficiency. The advantages of using ionic liquids instead of organic solvents as reaction media for biocatalysis include their ability to enhance enzyme activity as well as their selectivity and stability [6,7,8,9,13]. From an environmental point of view, the main advantage of ionic liquids is their lack of vapor pressure, which prevents the emission of solvent vapor into the atmosphere.

For many industrial applications, enzymes must be immobilized in order to increase their stability in operational conditions and allow their reuse. Among the different methods that can be used to immobilize enzymes, encapsulation is of particular interest because of its simplicity. The encapsulation process is based on entrapping the enzyme in a polymer matrix (gel), and there is no covalent association between the network and the enzyme [17]. Other advantages include the permeability of the matrices, which can be tuned to increase the selectivity of the biocatalyst. Very recently, de los Rios´ group have developed a new method for the enzymatic immobilization of laccase by entrapment in ionogel (a gel based on ionic liquids). Initially, (1-octyl-3-metylimidazolium bis(trifluoromethylsulfonyl)imide, cholinium bis(trifluoromethylsulfonyl)imide, cholinium dihydrogenphosphate, and hydroxyethylammonium formate were studied as choices for the constituents of the active phase of the ionogel. Using the new formulation, the enzyme’s activity and stability were dramatically improved, and when the new enzymatic derivatives were applied in a batch reactor to decolor the anthraquinonic dye Remazol Brilliant Blue R until 80% decoloration was obtained [18,19].

In this work, a new immobilization method was applied to *Candida antarctica* lipase B (CaLB) to synthesize butyl butyrate as a model reaction for the synthesis of esters. For this purpose, PVC (polyvinyl chloride) was used as polymer in which a suspension of the ionic liquids with the enzyme was immobilized by entrapment. As ionic liquid, 1-octyl-3-metylimidazolium hexafluorophosphate was used because it has been demonstrated to be a suitable reaction medium for the biocatalyst CaLB [20]. The activity at different temperatures and operational stability at high temperature of this new enzymatic ionogel were analyzed.

## 2. Materials and Methods 

### 2.1. Enzyme and Chemicals

A commercial lipase (EC 3.1.1.3) preparation was used as catalyst: free CaLB aqueous solution (lipozyme CaLB L), which was a gift from Novo España S.A. Polyvinyl chloride powder (PVC) was purchased from Sigma-Aldrich. 1-Octyl-3-methylimidazolium hexafluorophosphate ([Omim][PF_6_]) was purchased from IOLITEC (purity > 99%).

### 2.2. Immobilization of Free CaLB in Gels Based on Ionic Liquids (Ionogel)

**Figure 1:**
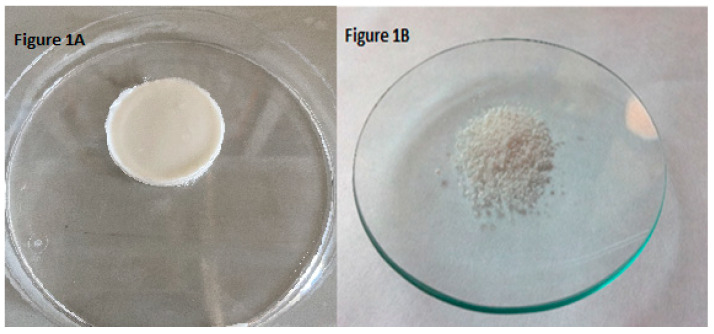
(**A**) Ionogel enzymatic derivative after casting, (**B**) Enzymatic derivative after crushing.

Free CaLB was entrapped by occlusion in an ionogel based on [Omim][PF_6_]. Derivatives of ionogel enzymes were prepared using 50% PVC and 50% IL using the following general procedure: 1 mL of THF was added to 200 µL of ionic liquid [Omim][PF_6_] and the mixture was stirred. Subsequently, 100 µL of a 14.2 mg/mL concentration CaLB solution or that four-fold diluted (3.55 mg/mL) in phosphate buffer (20 mM, pH = 7) was added to the IL mixture dissolved in THF under shaking conditions. Then, after adding 100, 20, or 0 µL of glutaraldehyde (25% water solution), 200 mg of PVC were added very slowly and continuously shaken. One additional milliliter of THF was added to allow the PVC solution. The process ended when the solution was seen to be homogeneous. The magnetic stirrer was removed, and the previous solution was poured into the center of an O-ring which was placed on a glass plate. The mixture was left to stand for 48 hours in a suction flow chamber to vaporize the THF while the PVC occluded the ionic liquid and enzyme it contained. After drying, the mixture was seen to solidify on the glass ring plate. The ring was then removed to release the circle of ionogel CaLB derivative, which was crushed for use in the reactors (Figure 1). The different enzymatic derivatives prepared are summarized in Table 1.

### 2.3. Ionogel-Derived Enzymatic Activity, Conversion, and Stability

**Figure 2:**
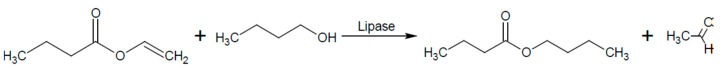
Synthesis of butyl butyrate from vinyl butyrate and 1-butanol catalyzed by CaLB.

Butyl butyrate synthesis from vinyl butyrate and butanol was used as reaction model (Figure 2) for the determination of activity, stability, and final conversion. For this, 0.5 mL (300 mmol) vinyl butyrate in hexane and 0.5 mL (300 mmol) 1-butanol in hexane were added to 2 mL screw-capped vials. The reaction was started by adding 80 mg ionogel CaLB derivative or free enzyme (1.42 or 0.355 mg protein) and allowed to run, whilst stirring, for 300 min at 30 °C. At different times, 30 µL aliquots were suspended in 920 µL hexane with 50 µL of valeric acid (100 mM, internal standard). The resulting solution (5 µL) was analyzed by GC. All experiments were carried out in duplicate, and the mean values are reported. The standard deviation was calculated in all cases. The efficiency of the catalytic action was measured based on two parameters: (**i**) the synthetic activity, defined as the amount of mmol of butyl butyrate produced per mg of protein and per minute and (**ii**) the final vinyl butyrate conversion at 300 min. One unit (U) produced 1 µmol of butyl butyrate per minute. Enzyme stability was measured by reusing the ionogel in different cycles at 70 °C. In each cycle the enzyme derivative was suspended in hexane for 24 h. For the stability assays the activity of CaLB was measured as described above and residual activity was calculated. Free CaLB (100 µL solution; 14.2 mg/mL) was also assayed in order to compare the efficiency of free enzyme with that of the immobilized enzymes.

### 2.4. Gas Chromatographic Analysis

The sample analyses were performed in a gas chromatograph (model 450 GC from Bruker, Germany) equipped with a flame ionization detector (FID), an autosampler injector, and a capillary column from J & W Scientific, Agilent Technologies, model HP INNOWAX, 30 m in length, 0.25 mm nominal diameter, and 0.25 micron film thickness. The injector temperature was 210 °C, working with a constant flow of 1 mL/min at the head of the column and split ratio of 1:10. N_2_ was used as carrier gas, while the temperature in the detector was 220 °C. The temperature program was as follows: 40 °C for 1 min; increase at 15 °C min^−1^ for 1 min, increase at 35 °C min^−1^ for 3 min; hold at 160 °C for 6 min. The retention times of the peaks were as follows: vinyl butyrate, 4.0 min; 1-butanol, 4.6 min; butyl butyrate, 5 min; butyric acid, 7.9 min; valeric acid (internal standard), 9.4 min. Substrate and product concentrations were calculated from calibration curves using stock solutions of pure compounds.

### 2.5. SEM–EDX Analysis

Scanning electron microscopy with energy dispersive X-ray spectroscopy (SEM–EDX) was applied for microimaging of the ionogel. The SEM images were acquired using a HITACH S-3500N apparatus following the protocol used by the SAIT platform (Scientific Instrumentation Service of the Technical University of Cartagena, Spain). The same equipment was used to generate the EDX profiles.

## 3. Results

### 3.1. Activity and Conversion of Ionogel-Derivatives

Among the ionic liquids used for enzyme-catalyzed reactions, water-immiscible ionic liquids were generally found to be suitable for biocatalytic reactions, while the water-miscible ionic liquids assayed were considered worse than conventional media. In the case of most water-miscible ILs [20], the negative effect observed on the lipase activity can be attributed to the direct interaction of the anion with the enzyme molecules, which would lead to protein denaturation or stripping off the essential water associated with the enzyme [21,22,23]. More specifically, van Rantwijk et al. [22] observed a strong coordinating effect on the part of anions, which led to the deactivation of CaLB in [Bmim][NO_3_], [Bmim][CH_3_CH_2_COO] and [bmim][dca], both of which are water-miscible ILs, and to which the dissolution of CaLB in these ILs is attributed. However, interaction between ILs and enzymes may sometimes improve enzyme behavior. The ionic liquid [Chol][H_2_PO_4_], which is water-soluble, was seen to be very effective at enhancing and stabilizing laccase activity, which was attributed to the modifications in the secondary structure of the enzymatic protein that it produced [24]. A modification of the secondary structure was also observed in CaLB in water-immiscible ionic liquids, which resulted in a more compact enzyme conformation that exhibited catalytic activity [25].

**Figure 3:**
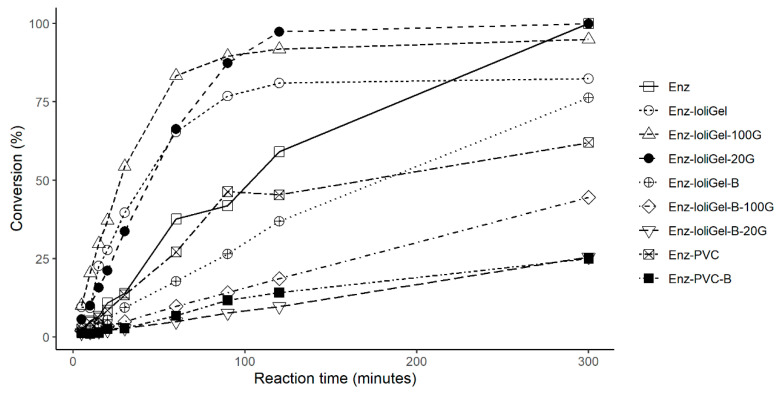
Time course of butyl butyrate synthesis from vinyl butyrate (150 mM) (not depicted) and 1-butanol (not depicted) (150 mM) at 30 °C. The synthesis activity was calculated from the initial slope and the conversion % was calculated at the end of the reaction (300 min).

As mentioned above, the present work describes how CaLB was entrapped in a polymeric matrix (PVC) with the ionic liquid 1-octyl-3-metylimidazolium hexafluorophosphate ([Omim][PF_6_]). The new enzymatic derivatives obtained were named ionogels of CaLB. In our assay, the hydrophobic ionic liquid, [Omim][PF_6_], was used because it creates a microenvironment that is suited to CaLB, thus increasing the enzyme activity over levels that can be obtained with hexane (used here as reference organic solvent) and other imidazolium-based ionic liquids with lower alkyl chain lengths that are commonly used as reaction media in transesterification reactions [7,14,26,27,28]. The entrapment method involving ionic liquids allows the enzyme to be immobilized by occluding CaLB within a suitable microenvironment that allows the enzyme to be retained while allowing the substrates and products of the reaction to cross the ionic liquid layer around the enzyme. Even if the ionic liquid is a good solvent for the substrates of the reaction, the microenvironment concentration of the substrates around the catalyst could be increased, consequently increasing the kinetics of the process. All enzyme derivatives (see Table 1) were tested as catalysts for the synthesis of the butyl butyrate from vinyl butyrate and 1-butanol. As a kinetically controlled reaction catalyzed by a serine hydrolase enzyme (CaLB), the transformation of vinyl butyrate is closely dependent on the nucleophile acceptors present in the reaction medium and involves competitive distribution of the rapidly formed acyl–enzyme intermediate between water (hydrolysis) or another nucleophile reagent, such as 1-butanol (transesterification). The latter synthetic pathway can be enhanced by using activated acyl donors such as vinyl esters with very low water content in the medium and high nucleophile (e.g., 1-butanol) concentration. The efficiency of the catalytic action can be measured by two parameters, the synthetic rate and the amount of converted vinyl butyrate measured at the end of the reaction (Figure 3). Free enzyme also was tested in order to compare the results with immobilized enzyme.

**Figure 4:**
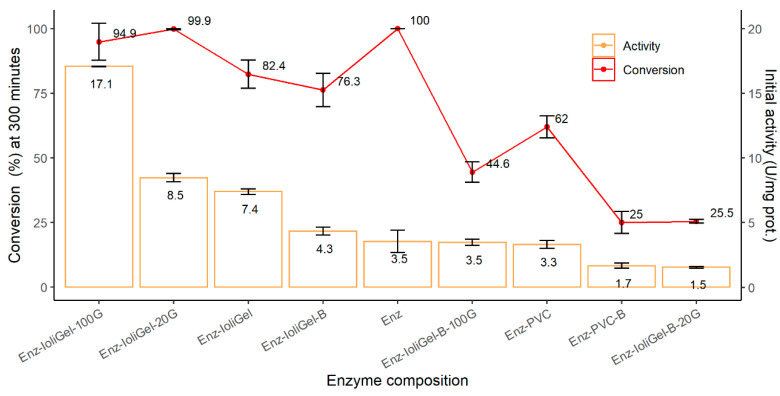
Activity and conversion exhibited by CaLB for butyl butyrate synthesis for different immobilized CaLB and free enzyme at 30 °C.

As can be seen from Figure 4, CaLB-ionogel containing [Omim][PF_6_] and 100 µL of glutaraldehyde had the best activity values, which are much higher than those achieved with the free enzyme. The second highest activity (half of the first one) was obtained when 20 µL glutaraldehyde was added. In the latter case (Enz-ionogel-20G), 100% conversion was reached at 300 min. Slightly lower activity (7.4 U/mg prot) was observed when neither glutaraldehyde nor buffer was added (Enz-ionogel). The activity of the free enzyme was much lower than the activity measured for the other CaLB-ionogel derivatives, except those produced with buffer and 20 µL of glutaraldehyde (Enz-ionogel-B-20G). It should be recalled here that “ionogel” in the reference name of the immobilized enzyme form stands for PVC+IL. The lowest activity value was that obtained when the diluted enzyme (with buffer) was immobilized in PVC without ionic liquid. By comparing the different activity and conversion values of the enzyme derivatives, it can be seen that the best activity and conversion values were obtained for the CaLB-ionogel derivative when enzymes undiluted (i.e., without buffer) were used: compare Enz-ionogel-B-100G vs. Enz-ionogel-100G, Enz-ionogel-B-20G vs. Enz-ionogel-20G, Enz-PVC-B vs. Enz-PVC). Hence, the higher the enzyme concentration in the enzymatic derivative, the higher the activity of the enzyme per mg of protein. Furthermore, increasing the amount of glutaraldehyde results in increasing enzyme activity: compare Enz-ionogel-100G vs. Enz-ionogel-20G and Enz-ionogel-B-100G vs. Enz-ionogel-B-20G, in which increasing the amount of glutaraldehyde used from 20 to 100 µL doubles the enzyme activity. This fact highlights the important role of glutaraldehyde in the enzymatic derivative. Sheldon developed a new method for enzyme immobilization as CLEA (crosslinked enzyme aggregates). The method involves the precipitation of the enzyme from aqueous buffer followed by crosslinking with glutaraldehyde of the resulting physical aggregates of enzyme molecules [29,30]. CLEAs are shown to be stable, recyclable catalysts exhibiting high catalyst productivities. For instance, *C. antarctica* lipase B, adsorbed and crosslinked on a polypropylene carrier, maintained its transesterification activity in the ionic liquids [BMIm][NO_3_] and [BMIm][dca], which deactivate the free enzyme [6]. Perhaps the formation of enzyme aggregates in ionogel derivatives by crosslinking with glutaraldehyde involves improvement of the enzymatic derivatives’ properties.

Note that the Enz-ionogel-100G reaches 5-fold higher activity than the free enzyme. It is also important to point out that in the case of Enz-PVC derivative, the reaction nearly stops at 40% conversion. It could be explained by possible PVC degradation at these conditions and, consequently, releasing HCl into the medium. Very recently, lipase degradation of plasticized PVC has been studied [29,30]. It was reported that this degradation could yield HCl. All the enzymatic derivatives, except Enz-PVC, are made of ionic liquids or contain buffer. The buffer could reduce the HCl concentration and the ionic liquids could reduce the effective HCl concentration. Therefore, the HCl could affect the enzyme activity only in the case of Enz-PVC. Furthermore, the ionogel could reduce PVC degradation.

In our previous work, laccase was immobilized in an ionogel based on 50% PVC and 50% the ionic liquid 1-octyl, 3-methyl imidazolium bistrifluoromethyl sulfonyl imide [Omim][NTf_2_]. To increase the activity and stability of the enzymatic derivative, glutaraldehyde was added at different concentrations (between 0.25% and 1.5%). An increase in activity and stability was observed at all the assayed concentrations, with the maximum being 0.5% glutaraldehyde [19]. In our experiments with CaLB, an increase in activity was observed at all assayed glutaraldehyde concentrations (between 1% and 4%) at 30 °C when the enzyme was used without dilution by buffer. The use of enzyme diluted with buffer decreased the activity with respect to non-diluted medium, probably because the diluted enzyme hindered crosslinking with glutaraldehyde. In the present work, the highest lipase activity was reached at 4% glutaraldehyde concentration without enzyme dilution, as mentioned. The effect observed for glutaraldehyde in both cases (laccase and lipase) shows the importance of the crosslinking to enhance the activity of the immobilized derivative.

We can also observe that the immobilization of the enzyme on a PVC support without ionic liquid provided the lowest activity values. Furthermore, the comparison of enzymatic derivatives with and without ionic liquids (i.e., Enz-PVC-B vs. Enz-ionogel-B and Enz-PVC vs. Enz-ionogel) demonstrates the importance of using ionic liquid in the preparation of the immobilized derivatives. As mentioned above, the increase in activity could be due to the suitable microenvironment created by the ionic liquid around the enzyme and/or to increased substrate concentration in the ionic liquid microenvironment around the enzyme. In this immobilization method, the enzyme is entrapped by the ionic liquid and PVC, and the ionic liquid could absorb substrates, increasing the substrate concentration in the microenvironment of the enzyme. In this respect, the distribution ratio of vinyl butyrate, butanol, and butyl butyrate between ionic liquid and hexane was studied by Hernández-Fernández et al. 2010 [2]. The distribution ratio of vinyl butyrate, butanol, and butyl butyrate between [Omim][PF_6_] and hexane was 0.68, 3.63, and 0.35, respectively. These distribution ratios could increase the concentration of butanol in the microenvironment of the enzyme and remove the product butyl butyrate from this microenvironment. All the above might improve the kinetics of the bioreaction. Moreover, it has been demonstrated [12] that enzyme immobilization involves more stable enzymes in exchange of less enzymatic activity. As mentioned above, the major advantage of this method is that it allows more active derivatives to be created than in the case of free enzyme, as can be seen observed from Figure 4.

Regarding the selectivity of the reaction, the byproduct butyric acid was not observed in the enzymatic derivatives obtained using ionic liquids. The synthetic pathway was enhanced by the use of activated acyl donors (vinyl butyrate), a medium with a very low water content (hexane), and a strong nucleophile (1-butanol). Furthermore, the ionic liquid has a specific ability to reduce water activity in the enzyme microenvironment by capturing the free molecular water and, consequently, reducing the hydrolysis reaction. The increased selectivity of transesterification reactions using ionic liquids has been previously described [9,20].

As regards the greenness of the process, we should remember that the primary cause of the high E factors (developed by Seldon and defined as the ratio mass waste/mass product) of most processes in the pharmaceutical industry is the high molecular complexity of APIs (active pharmaceutical ingredients), the large number of chemical steps needed to assemble the APIs from commercially available starting materials, and the use of classical stoichiometric reagents instead of catalysts [29,31]. From an environmental point of view, the new method proposed in this paper involves the use of immobilized biocatalysts with better properties, activity, and selectivity than the free enzyme, which increases the productivity of the process [30,32]. Furthermore, the ionic liquid that traps the enzyme not only improves the catalytic activity of the biocatalyst but also concentrates the substrate around the catalyst, increasing the kinetics of the reaction. The ionic liquid around the catalyst was also able to acts as a selective membrane between the medium and the biocatalyst, regulating the transport of substrates towards the catalyst and, consequently, improving the overall selectivity of the process. The consequence of all this is that number of the purification steps could be reduced, thereby making the whole process greener.

### 3.2. Stability of Ionogel Enzymatic Derivatives

**Figure 5:**
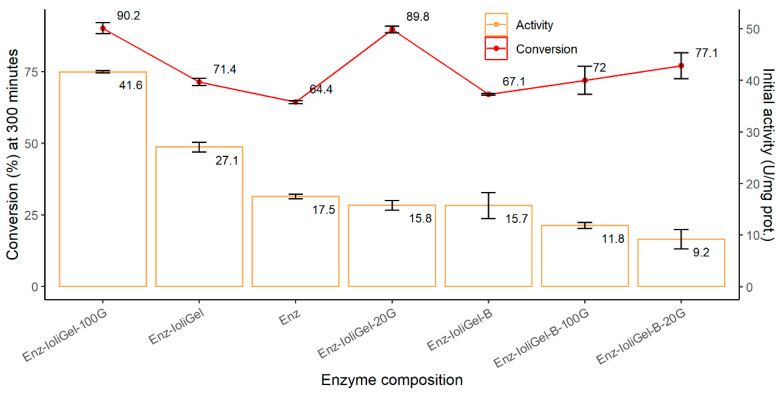
Activity and conversion exhibited by CaLB during butyl butyrate synthesis using different immobilized CaLB (ionogel) and free enzyme at 70 °C.

The stability of the new enzymatic derivatives was measured by reusing the catalyst in 5-day cycles. Figure 5 shows the activity of the first cycle using the ionogel of CaLB and the free enzyme at 70 °C. The relative activity between the different immobilized derivatives was similar at 30 °C, which corroborates the results found for the new enzymatic derivatives. Furthermore, in all cases, the activity increased two- to three-fold by increasing temperature because the kinetic constant increased with temperature. However, the conversion was higher in some cases and lower in others—while higher initial activity could lead to a higher final conversion %, a higher temperature might also involve lower final conversion % due to deactivation of the enzyme. It is important to point out that in nearly all cases, the activity of the undiluted immobilized enzyme was higher than that of the free enzyme. Furthermore, at 70 °C, it was observed that using the concentrated enzyme in the immobilized derivative led to better activity and final conversion values than when the enzyme diluted in phosphate buffer was used. The same behavior was observed with increasing concentrations of glutaraldehyde, since activity was higher with 100 µL than with 20 µL of glutaraldehyde. As mentioned above, the same effect of glutaraldehyde concentration on the stability of immobilized laccase was observed in a previous work, when maximum stability was found at a 0.5% glutaraldehyde concentration [19].

**Figure 6:**
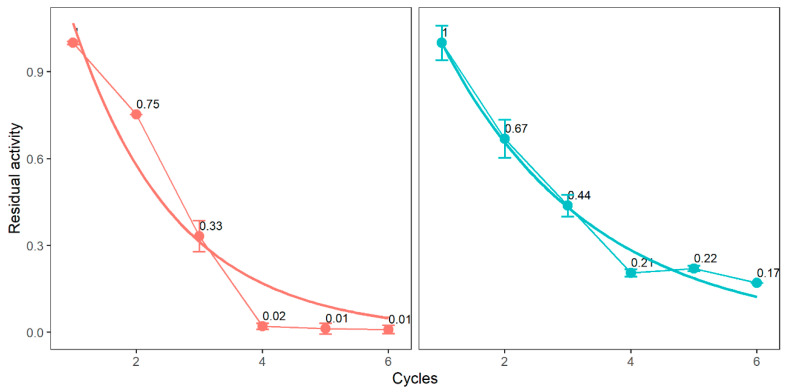
Deactivation profile of ENZ-ionogel-20,100G in hexane at 70 °C.

To analyze the operational stability of the new enzyme derivatives at a high temperature (70 °C), six cycles of 24 h each were carried out using the same enzyme. Only the ionogel composed of concentrated enzyme (without buffer) and 100 or 20 µL of glutaraldehyde remained active after the first cycle at 70 °C, while the rest of the enzyme derivatives were inactivated after the first assay. Figure 6 depicts the stability of Enz-ionogel-20,100G vs. temperature, represented as a decay of the initial activity. The half -life of the ionogel with 100 µL glutaraldehyde and with 20 µL glutaraldehyde was similar for around 3 cycles, which means for 60–72 h. The half-life was calculated for these two derivatives considering that each cycle lasted 24 h. 

As example, Jiang et al. (2009) immobilized *Candida rugosa* lipase on magnetic nanoparticles supported ionic liquids with different cation chain lengths and anions, Cl^−^, BF_4_^−^, and PF_6_^−^ [10]. The activity of bound *Candida rugosa* lipase was higher (118.3%) than that of the native lipase. The activity of *Candida rugosa* lipase immobilized on magnetic nanoparticle-supported ionic liquids reached a maximum at 37 °C, after which it decreased, remaining at a constant 60% when the temperature reached 80 °C. For ionogel-immobilized CaLB, as mentioned above, the activity at 70 °C increased two- to three-fold over the activity observed at 30 °C, which was explained by the increasing kinetic constant with temperature. *Candida rugosa* lipase retained 60% of its initial activity after eight batch reactions. Considering that each cycle lasted 5 hours, the lipase retained 60% of its activity at 40 h. As mentioned above, similar results were obtained with some ionogel CaLB derivatives, with the half-life of the ionogel with 100 and 20 µL glutaraldehyde being around 60–72 h. Another example related to CaLB analyzed the enzyme immobilized by adsorption onto 12 different modified silica supports with different alkyl chain lengths and functional groups. Immobilized enzyme particles were coated with ionic liquids (butyltrimethylammonium bistriflimide or trioctylmethylammoniun bistriflimide) by adsorption. The immobilized derivatives were assayed for the kinetic resolution of *rac*-1-phenylethanol in both ionic liquid/hexane and ionic liquid/supercritical carbon dioxide biphasic media. The best results were obtained for the supports modified with non-functionalized alkyl chains. The activity of the immobilized enzyme particles coated with ionic liquids was lower in hexane than when using the immobilized enzyme with no such coating, which was explained by the stronger mass transfer limitation caused by the ionic liquids. However, half-life times were enhanced in hexane media at 95 °C [31,33]. Porcine pancreatic lipase (PPL) was also immobilized using ionic liquids for that magnetic chitosan nanocomposites modified with a functional imidazolium-based IL. The activity of almost every PPL derivative was higher than that of the free enzyme when working above 50 °C. The thermal stability of the PPL derivatives at 50 °C ranged from 45% to 80% at 6 h [14].

### 3.3. SEM–EDX Characterization of the Ionogel Enzymatic Derivative

The ionogel enzymatic derivatives, which are made of ionic liquid 1-octyl-3-methylimidazolium hexafluorophosphate, PVC, and enzyme, were analyzed by SEM–EDX, after and before being crushed, and after and before being used.

**Figure 7:**
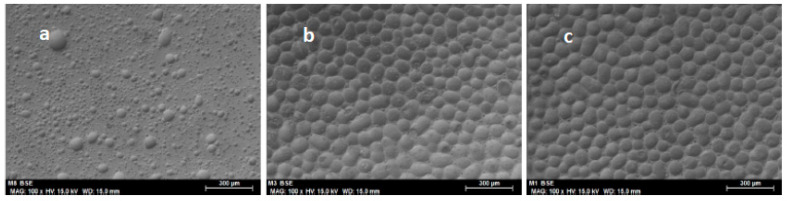
Scanning electron micrographs of the ionogel enzymatic derivative without crushing: (Enz-ionogel-B) (100×): (**a**) fresh (scale bar = 300 μm), (**b**) after 1 cycle (scale bar = 300 μm), and (**c**) after 5 cycles (scale bar = 300 μm).

Figure 7 depicts SEM micrographs of samples with buffer-diluted enzyme (without crushing) (Enz-ionogel-B), fresh and after 1 and 5 reaction cycles (24 h each) at 30 °C. Notable morphological differences can be observed between the fresh ionogel enzymatic derivative (Figure 7) and the same ionogel enzymatic derivative after 1 and 5 reaction cycles (Figure 7b,c, respectively). The irregularly distributed grains of Figure 7a change completely to show well-defined grains at the end of the first cycle (Figure 7b), a morphology that is maintained at the end of the 5 cycles (Figure 7c). This change in morphology might be due to the interaction of the PVC in the ionogel with the hexane reaction medium, giving rise to the “bubbles” that practically cover the surface of the ionogel as soon as it is used.

**Figure 8:**
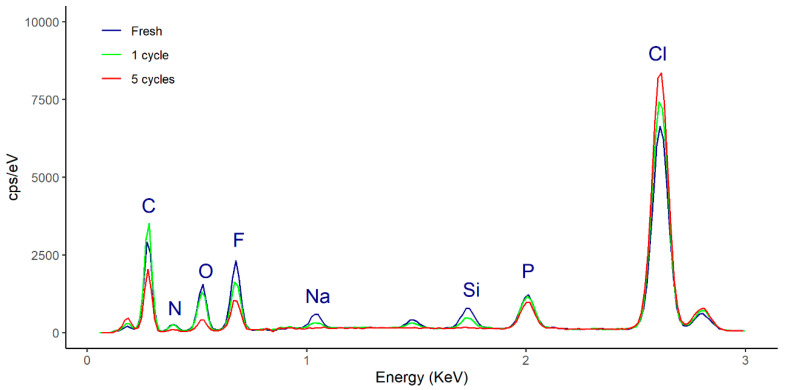
EDX spectra of the ionogel enzymatic derivative (Enz-ionogel-B): fresh (blue line), after 1 cycle (green line), and after 5 cycles (red line).

As regards the composition of the fresh ionogel enzymatic derivative (without crushing), the EDX spectrum (Figure 8) shows that it has maintained its stability during all the operation cycles, as is evident from the superimposed peaks of the representative elements (peak F represents [Omim][PF_6_] and peak Cl represents PVC). It should be noted that the concentration of fluorine measured in the selected samples increases with the number of reaction cycles, from 6.59% (fresh) to 7.94% (1 cycle) and, finally, 11.51% (5 cycles). This increase suggests that the IL in the ionogel migrates outward from it during the course of the reaction. It should be noted that EDX provides information on the composition of elements at a sampling depth of 1–2 μm.

The SEM–EDX spectra of ionogel prepared with glutaraldehyde (Enz-ionogel-B-20,100G) were also analyzed. The glutaraldehyde was added to the ionogel in order to achieve crosslinking of the enzyme units by allowing the functional groups of the glutaraldehyde to react with the free amino groups of the enzymes, thus forming Schiff bases. This crosslinking provides stability to the enzyme derivative, as mentioned above.

**Figure 9:**
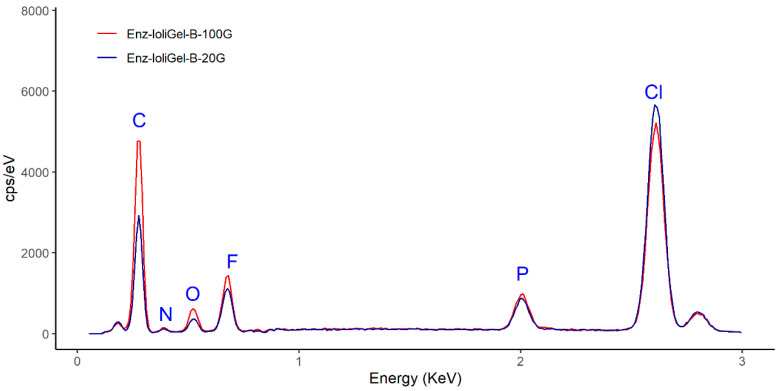
EDX spectra of the ionogel enzymatic derivative (Enz-ionogel-B-20,100G): 100 μL glutaraldehyde (red line) and 20 μL glutaraldehyde (blue line).

**Figure 10:**
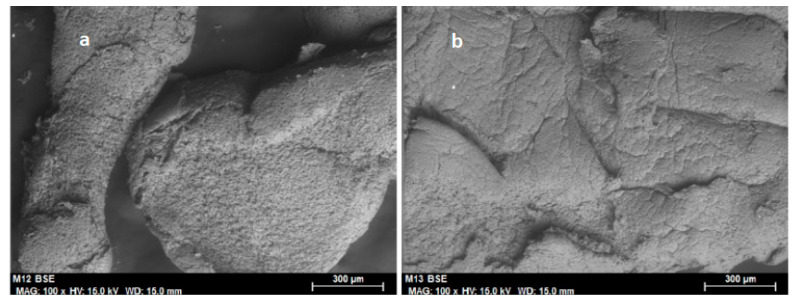
Scanning electron micrographs of the ionogel with diluted enzyme incorporating glutaraldehyde (25% aqueous solution) enzymatic derivative (Enz-ionogel-B-20,100G): (**a**) 100 μL glutaraldehyde (100×) (scale bar = 300 μm) and (**b**) 20 μL glutaraldehyde (100×) (scale bar = 300 μm).

Figure 9 shows the EDX spectra corresponding to fresh ionogel with diluted enzyme incorporating glutaraldehyde in the form of a 25% aqueous solution (20 or 100 μL). It can be seen that the carbon peak was significantly higher in the ionogel with 100 μL of glutaraldehyde than when 20 μL was used. Comparing the SEM micrographs of both ionogel enzymatic derivatives (Figure 10a,b), it can be seen than the ionogel enzymatic derivative made with 100 μL is slightly more porous than with 20 μL. This circumstance favors an increase in catalytic activity, due to the greater active contact surface between the enzyme and substrates. Thus, the higher the concentration of glutaraldehyde, the greater the crosslinking and porosity and, consequently, the greater the specific activity and conversion achieved. This is corroborated by comparing the activities of the respective ionogels. In the first case, the specific activity reached was 3.5, and in the second, 1.5, with respective conversions of 45% and 26%. In a previous work with laccase, the group led by de los Rios proposed that the activity and stability of the enzyme are related to the carrier’s porosity and the hydrophobicity of the ionic liquids [8]. Also, a very recent work demonstrates that the addition of glutaraldehyde to the mixture of laccase–PVC–ionic liquid could contribute not only to the crosslinking of the enzyme but also to modification of the macrostructure of the membrane surfaces [19].

However, when ionogel with buffer and without glutaraldehyde was used, the activity was higher than in the case of ionogel with buffer and glutaraldehyde (20 or 100 μL). It can be hypothesized, in this case, that the addition of glutaraldehyde in the presence of a buffer provides an “extra” degree of dilution to the enzyme, which would harm the enzymatic activity. It is important to point out that the ionogel was crushed before use. When ionogels are crushed (Figure 10a,b) their appearance changes (Figure 7a, without crushing). Before use, the ionogels were crushed with a grinder, which might cause an increase in temperature and major surface restructuring due to this thermal stress. 

**Figure 11:**
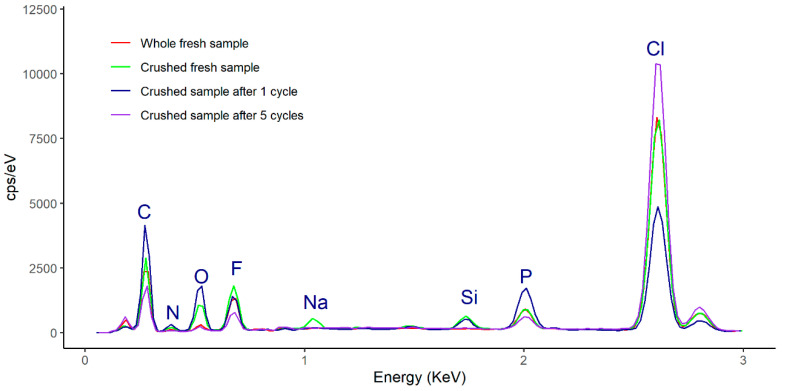
EDX spectra of the sample prepared with PVC and ionic liquids [Omim] [PF_6_] in the absence of enzyme: whole fresh sample (red line), crushed fresh sample (green line), crushed sample after 1 cycle (blue line), and crushed sample after 5 cycles (purple line).

Samples prepared only with PVC and ionic liquid and without enzyme were analyzed both fresh and after 5 cycles of operation. Figure 11 compares the EDX spectra of the whole and crushed fresh samples. There is practically complete overlapping of peaks, except for chlorine, which is more abundant in the whole sample, perhaps because of accumulation on some surfaces. The most relevant aspect is the total homogeneity that can be observed in the SEM micrographs (Figure 11), which do not show any relief in the intact (Figure 11 blue line) or crushed (Figure 11 green line) samples. This completely smooth surface was only observed in the absence of enzyme, when its presence makes the membrane appreciably more porous, as can be seen in Figure 7a. This may be due to water being introduced with the enzyme solution.

**Figure 12:**
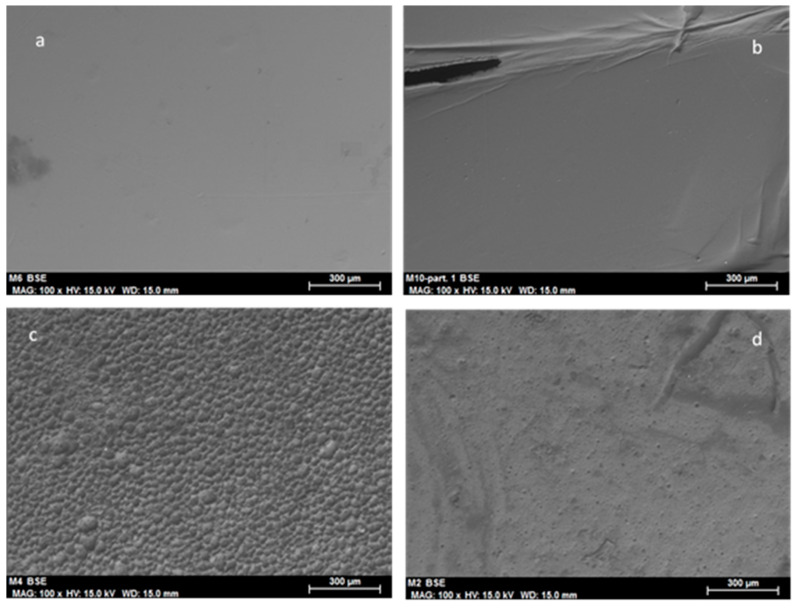
Scanning electron micrographs of the sample prepared with PVC and ionic liquids [Omim] [NTf_2_] in the absence of enzyme: (**a**) whole fresh sample (100×) (scale bar = 300 μm), (**b**) crushed fresh sample (100×) (scale bar = 300 μm), (**c**) crushed sample after 1 cycle (100×) (scale bar = 300 μm), and (**d**) crushed sample after 5 cycles (100×) (scale bar = 300 μm).

Figure 11; Figure 12 depict study the stability of the sample made of PVC and [Omim] [PF_6_] as measured by SEM–EDX. Note that the proportion of ionic liquid (fluorine peak) decreases significantly from the fresh sample (Figure 11, 25.41%) to the samples used for 1 cycle (Figure 11, 7.73%) and 5 cycles (Figure 11, 9,16%). The loss of IL when enzyme is not used in the formulation clearly contrasts with the increased peak observed for the ionogel with enzyme (Enz-ionogel-B, see Figure 8). The explanation lies in the increase in hydrophilicity of the IL (which is normally hydrophobic) when the enzyme derivative is incorporated dissolved in water, thus reducing the possibility of migration to the hexane (hydrophobic) reaction medium. The corresponding SEM micrographs (Figure 12) show the superficial change that takes place in the samples, such as the fresh sample (Figure 12a,b). As can been observed, the smooth surface (Figure 12a,b) changes completely to show well-defined grains (Figure 12c,d). A similar phenomenon occurred with the ionogel enzymatic derivatives (see Figure 7), which was due to hexane causing the PVC to swell.

## 4. Conclusions

A new method for immobilizing enzymes by entrapment in a gel based on an ionic liquid (ionogel) is described for CaLB. The use of the CaLB-ionogel based on the ionic liquid [Omim] [PF_6_] and the organic polymer PVC led to higher enzymatic activities than when free CaLB was used. The use of glutaraldehyde as crosslinker agent improved the activity of the enzymatic derivative, with the highest activity being reached with PVC, [Omim][PF_6_] ionic liquid, CaLB, and 100 μL of glutaraldehyde. The operational stability of all the enzymatic derivatives (named CaLB-ionogel) was tested at high temperatures (70 °C). Only the CaLB-ionogels prepared with glutaraldehyde were still active after 2 cycles (24 hour each cycle). The porosity of the catalyst was found to be a key factor for the activity of the enzyme. Furthermore, the ionic liquid used could play a double role: on the one hand, creating a suitable microenvironment for the enzyme, thereby increasing its activity and stability and, on the other hand, increasing the substrate concentration in the enzyme microenvironment by concentrating in the ionic liquids, thus avoiding limitations in mass transfer and increasing the kinetics of the reaction. If the ionic liquids are correctly chosen, the ionic liquids could also act as a selective liquid membrane around the biocatalyst for different substrates, improving the overall selectivity of the process. All mentioned properties demonstrate that the new biocatalyst makes processes greener. The results of this study are quite encouraging and suggest a new way to easily design a biocatalyst. By changing the ionic liquid that was used, the activity, and even the selectivity of the catalyst could be modified. For instance, to increase the activity and selectivity of a given biocatalyst for a specific substrate, it is simply a matter of choosing an ionic liquid that makes a suitable microenvironment for the enzyme and, also, in which the specific substrate will be preferentially absorbed. The method increases the potential field of application for existing biocatalysts.

## Figures and Tables

**Table 1:** Chemical composition of the prepared ionogel enzymatic derivatives. The “ionogel” in the name of the immobilized enzyme stands for PVC + IL.

	Enzyme (µL)	Ionic Liquid [Omim][PF_6_] (mg)	PVC (mg)	Glutaraldehyde 25% in Water (µL)	[Protein in the Reactor], 80 mg of Ionogel Was Used
**Enz ^a^**	100 (14.2 mg prot/mL)	0	0	0	1.420
**Enz-PVC ^b^**	100 (14.2 mg prot/mL)	0	200	0	0.379
**Enz-PVC-B ^c^**	100 (3.55 mg prot/mL) *	0	200	0	0.095
**Enz-ionogel ^d^**	100 (14.2 mg prot/mL)	200	200	0	0.227
**Enz-ionogel-B ^e^**	100 (3.55 mg prot/mL) *	200	200	0	0.057
**Enz-ionogel-20G ^f^**	100 (14.2 mg prot/mL)	200	200	20	0.218
**Enz-ionogel-100G ^g^**	100 (14.2 mg prot/mL)	200	200	100	0.189
**Enz-ionogel-B-20G ^h^**	100 (3.55 mg prot/mL) *	200	200	20	0.055
**Enz-ionogel-B-100G ^i^**	100 (3.55 mg prot/mL) *	200	200	100	0.047

* Initial enzyme concentration diluted four-fold with phosphate buffer., ^a^ Free Enzyme., ^b^ PVC enzymatic derivative without IL., ^c^ PVC enzymatic derivative with buffer (B) and without IL., ^d^ Ionogel enzymatic derivative without buffer ^e^ Free Enzyme., ^e^ Ionogel enzymatic derivative and buffer., ^f^ Ionogel enzymatic derivative and 20 µL glutaraldehyde and without buffer., ^g^ Ionogel enzymatic derivative and 100 µL glutaraldehyde and without buffer., ^h^ Ionogel enzymatic derivative and 20 µL glutaraldehyde and with buffer., ^ij^ Ionogel enzymatic derivative and 100 µL glutaraldehyde and with buffer (B).
